# Colonic and Hepatic Modulation by Lipoic Acid and/or N-Acetylcysteine Supplementation in Mild Ulcerative Colitis Induced by Dextran Sodium Sulfate in Rats

**DOI:** 10.1155/2016/4047362

**Published:** 2016-11-13

**Authors:** Fabiana Andréa Moura, Kívia Queiroz de Andrade, Orlando Roberto Pimentel de Araújo, Valéria Nunes-Souza, Juliana Célia de Farias Santos, Luiza Antas Rabelo, Marília Oliveira Fonseca Goulart

**Affiliations:** ^1^Faculdade de Nutrição (FANUT), Universidade Federal de Alagoas (UFAL), Campus A. C. Simões, Tabuleiro dos Martins, 57072-970 Maceió, AL, Brazil; ^2^Pós-Graduação em Ciências da Saúde (PPGCS/UFAL), Maceió, AL, Brazil; ^3^Instituto de Química e Biotecnologia (IQB/UFAL), Maceió, AL, Brazil; ^4^Laboratório de Reatividade Cardiovascular (LRC/UFAL), Maceió, AL, Brazil; ^5^Departamento de Fisiologia e Farmacologia (DFF), Centro de Biociências (CB), Universidade Federal de Pernambuco (UFPE), Av. Prof. Moraes Rego, 1235 Cidade Universitária, 50670-901 Recife, PE, Brazil; ^6^Rede Nordeste de Biotecnologia (RENORBIO), Recife, PE, Brazil

## Abstract

Lipoic acid (LA) and* N*-acetylcysteine (NAC) are antioxidant and anti-inflammatory agents that have not yet been tested on mild ulcerative colitis (UC). This study aims to evaluate the action of LA and/or NAC, on oxidative stress and inflammation markers in colonic and hepatic rat tissues with mild UC, induced by dextran sodium sulfate (DSS) (2% w/v). LA and/or NAC (100 mg·kg·day^−1^, each) were given, once a day, in the diet, in a pretreatment phase (7 days) and during UC induction (5 days). Colitis induction was confirmed by histological and biochemical analyses (high performance liquid chromatography, spectrophotometry, and Multiplex®). A redox imbalance occurred before an immunological disruption in the colon. NAC led to a decrease in hydrogen peroxide (H_2_O_2_), malondialdehyde (MDA) levels, and myeloperoxidase activity. In the liver, DSS did not cause damage but treatments with both antioxidants were potentially harmful, with LA increasing MDA and LA + NAC increasing H_2_O_2_, tumor necrosis factor alpha, interferon gamma, and transaminases. In summary, NAC exhibited the highest colonic antioxidant and anti-inflammatory activity, while LA + NAC caused hepatic damage.

## 1. Introduction

The use of antioxidants is considered an important complementary therapy in several conditions such as cardiometabolic, neurological, and gastrointestinal (GI) diseases, for cancer prevention and others [1]. Among the GI disorders, inflammatory bowel diseases (IBD), composed of ulcerative colitis (UC) and Crohn's disease (CD), affect 150–250/100,000 people, especially in the USA and Europe, with substantial health care costs of approximately US$6 billion and €4.6–5.6 billion, per year, respectively [2, 3].

Despite extensive research, the etiology of IBD has yet to be elucidated; however it is considered closely connected to genetic and immunologic factors, microbiota, and oxidative stress, which, in the case of UC, concerns both etiology and symptoms such as increase of intestinal permeability by destruction of tight junctions and increase of infection and inflammation by neutrophil infiltration. Hence, the presence of colonic ulcers, as a consequence of lipid peroxidation and protein damage and development of cancer, is due to DNA damage [4]. However, the cause of these symptoms and clinical manifestations is heterogeneous, varying according to the UC level: mild, moderate, or severe. In humans, the most common and problematic stage of UC is mild colitis [5]. It is characterized by normal albumin, body temperature, pulse, and hematocrit ratio, associated with an erythrocyte sedimentation rate of <20 mm/h, less than 4 bowel movements per day and no weight loss [6]. Nevertheless, this classification may differ according to authors and adopted criteria [7, 8].

The extraintestinal manifestations of IBD, such as the systemic effects of alternative therapy used for IBD treatment, are poorly explored. Among these manifestations, emphasis is given to hepatobiliary disorders. The close relationship between the liver and intestine is justified by their common embryogenesis until later in adult life (intestine to portal vein) [9]. However, the main focus of the cited studies was microbiota, which has received special attention due to its intimate connection with metabolic syndrome, obesity, and nonalcoholic fatty liver diseases [10].

A pioneer study showed that, after colitis induction (4% of DSS for 7 days), important liver injury occurred, confirmed by higher serum levels of haptoglobin and various histological findings, such as signs of necrosis and ballooning of hepatocytes [11].

The use of antioxidants has emerged as an alternative therapy for IBD. Among them, lipoic acid (LA) and* N-*acetylcysteine (NAC) stand out, which have been tested separately in UC [4], and in combination for other clinical conditions [11, 12], with positive results.

Due to its antioxidant property in both forms, oxidized (LA) and reduced (dihydrolipoic acid, DHLA), LA is called a “universal antioxidant.” The LA/DHLA couple is a scavenger of superoxide anion radical (O_2_^•−^), hypochlorous acid (HOCl), peroxynitrite (ONOO^−^), and nitric oxide (^•^NO). It is able to restore the exogenous antioxidants tocopherol and ascorbic acid and the endogenous antioxidant system of reduced/oxidized glutathione (GSH/GSSG) [6]. Additionally,* in vitro* studies suggest that LA acts as an inhibitor of I*κ*B kinase-2 (IKK2), with subsequent release of nuclear factor kappa B (NF-kB) [13]. Another anti-inflammatory effect is the elevation of nuclear factor (erythroid-derived 2)-like 2 (Nrf2) intracellular levels that occurs through independent mechanisms, by breaking links between Nrf2 and Keap 1 (Kelch ECH associating protein 1). These actions lead to decreased expression of various proinflammatory cytokines and increased expression of enzymatic antioxidants, such as glutathione peroxidase (GPx) and superoxide dismutase (SOD) [14].

NAC is a strong reducing agent [15]. Like LA, NAC is an important scavenger of reactive molecules and plays a role as a metal chelator. However, the most important antioxidant action assigned to NAC is the increase in antioxidant defense activity by providing cysteine, which is required for GSH synthesis. The anti-inflammatory effects of NAC have also been confirmed, through observation of NF-kB inhibition [16].

Taking into consideration that LA and NAC, alone and in combination, were successfully tested in several systems and in regard the intimate bowel-liver association, the aim of this study was to evaluate the action of LA and/or NAC, on oxidative stress and inflammation in mild colitis induced by DSS.

## 2. Materials and Methods

### 2.1. Chemicals

DSS MW 36,000–40,000 Da is from MP Biomedicals. LiChrolut®, RP-18 E is from Merck, cytokine kits are from Milliplex MAP Mouse Cytokine/Chemokine Panel (Merck Millipore®), protease inhibitor cocktail tablets are from Roche®, and RIPA buffer are from Cell Signaling®. LA and NAC from Ao Pharmaceutico (Alagoas/Brazil) and all other chemicals and enzymes from Sigma-Aldrich® Chemicals.

### 2.2. Equipment

HPLC coupled to UV detector (Shimadzu®), freezer VIP Series Sanyo, spectrofluorometer (ThermoScientif® Multiskan), and an optic microscopy Olympus BX51 attached to a DP70 Digital Camera System and a MAGPIX® Multiplex Reader were used.

### 2.3. Animals

Experimental protocol was approved by the Institutional Animal Ethic Committee/Universidade Federal de Alagoas (IAEC/UFAL) (05/2014) and was performed in accordance with the Committee for the Purpose of Control and Supervision of Experimentation on Animals (CPCSEA) guidelines. Adult male Wistar rats, 185–203 g, were obtained from the Central Animal House of UFAL and kept in individual cages, under controlled conditions, at room temperature (22 ± 2°C), humidity (50 ± 10%), and light (12/12 h light/dark). Commercial feed (Nuvilab®) and water were provided* ad libitum*.

### 2.4. UC Induction and Experimental Design

Animals were divided into 5 groups. Healthy rats received commercial feed (control group). Mild UC was induced in four groups with 2% w/v DSS, administered in drinking water for 5 days. Antioxidants-treated rats were supplemented 7 days before mild colitis induction with lipoic acid (LA, 100 mg/kg/d), N-acetylcysteine (NAC, 100 mg/kg/d), or LA + NAC (100 mg/kg/d, each). Doses of the two antioxidants used are considered safe to rats/mice (LA up to 1800 mg/d [17] and NAC up to 6000 mg/d) and have been tested successfully in several physiological/pathophysiological conditions in mice/rats [16, 18].

### 2.5. Euthanasia, Blood Sample Collection, General Biochemical Profile, and Preparation of Tissue Homogenates

At the 13th day, after 12 h of fasting, the animals were anesthetized (ketamine, 100 mg/kg, and xylazine, 15 mg/kg, via i.p.), and blood was collected by cardiac puncture. After perfusion with heparine solution (1 : 50 v/v), aorta was sectioned. The organs were, then, dissected, placed on liquid nitrogen, and immediately stored at −80°C. Tissue homogenates were prepared, within ice cold, with Ripa buffer with protease inhibitor cocktail (one tablet for 50 mL of Ripa buffer), and were centrifuged at 19.600 ×g/20 min, at 4°C. The supernatants were stored at −80°C.

Biochemical analyses were made by certified laboratory, using well-established methods. Colon and liver tissues were washed in saline and weighed. The left lobe of the liver and distal colon were cut, for use in histological analysis.

### 2.6. Histological Analysis

After fixation with 10% buffered formalin, the organs were cleaved and the sections were processed by embedding in paraffin and stained with hematoxylin and eosin (HE) to histological evaluation.

Total collagen was evaluated by Masson trichrome staining kit. Briefly, one section of each, liver and bowel, was obtained and stained by using Masson's original trichrome stain, with collagen stained in blue, nuclei in purple-brown, and cytoplasm in pink. Collagen area was defined as the distinct blue color region and was distinguished from muscle, blood, and inflammatory cells. Afterwards, ImageJ® software was used to quantify the blue area (pixel/camp).

### 2.7. Malondialdehyde (MDA), Hydrogen Peroxide (H_2_O_2_), and Nitrite Levels

MDA contents were measured by reverse phase ion pair HPLC, with UV detector at 270 nm, according to Tatum et al. [19]. HPLC system conditions were C18, ultrasphere column 150 mm × 4.6 mm and 45 mm × 4.6 mm guard column, the mobile phase comprised acetonitrile HPLC/UV grade, and Trizma buffer (pH 7.4) (1 : 9). The intestinal or hepatic tissue was homogenized with Trizma buffer, BHT, and acetonitrile. Then, the homogenate was centrifuged at 872 ×g/10 min, at 4°C, and the supernatant was filtrated with HPLC filter (0.22 mm). The flow rate was 1.0 mL·min^−1^ and MDA was calculated from the standard curve, generated using 1,1,3,3-tetramethoxypropane and expressed as nmol MDA·mg tissue^−1^. The retention time was around 2 min 40 s.

H_2_O_2_ was measured according to Pick and Keisari [20]. Hepatic or colonic tissues were homogenized in 1.0 mL of assay buffer containing 140 mM NaCl, 10 mM PBS, pH 7.0, and 5.5 mM dextrose. After centrifugation (2000 ×g/5 min at 4°C), the supernatant was transferred to microtubes, containing 0.28 mM phenol red, 8.5 U/mL of horseradish peroxidase, and assay buffer. After incubation (37°C for 30 min), NaOH 1 M was added. The samples were read at 610 nm. The concentration was expressed as nmol·mg protein^−1^.

Nitrite assay was performed based on Griess method adapted for microplates. Supernatant was mixed with 2,3-diaminonaphthalene (50 *μ*g·mL^−1^ in 0.2 M HCl) and incubated at room temperature/10 min. The reaction was interrupted by the addition of NaOH (2.8 M) and monitored at 540 nm. The result was expressed as *μ*mol·mg protein^−1^.

### 2.8. Measurement of Enzyme Activity

SOD was measured following S. Marklund and G. Marklund [21]. Liver or colon supernatant was added to 0.2 mM pyrogallol (dissolved in 50 mM potassium phosphate buffer (PBS), pH 6.5) to initiate the reaction, and the decrease of the absorbance related to pyrogallol was monitored at 420 nm. One unit of SOD was defined as the amount required for inhibiting pyrogallol autoxidation by 50% per min. The result was expressed as U·mg protein^−1^ [22].

CAT activity was measured as the rate of decomposition of H_2_O_2_, as described elsewhere [23], and was monitored at 540 nm. Relative activity was expressed as IU·min·mL·mg protein^−1^.

Total GPx activity was measured according to Flohe and Gunzler [24], adapted to microplate. The hepatic or colonic tissue was homogenized with assay buffer (PBS 0.1 M, EDTA 5 mM, pH 7.4) and centrifuged (14.000 ×g/20 min, at 4°C). Supernatant was added to wells (in duplicate), followed by the addition of glutathione reductase (GR) (0.048 U) and GSH (10 mM), incubated at 37°C/10 min and afterwards, nicotinamide adenine dinucleotide phosphate (NADPH) (1.5 mmol) and* tert*-butyl hydroperoxide (0.5 mM) were added. The decrease in the absorbance of the system was measured, for 5 min, at 340 nm. One unit of tGPx was defined as the amount of enzyme able to catalyze the oxidation of 1 *μ*mol of NADPH to NADP^+^ in 1 min. The result was expressed as U·mg protein^−1^.

MPO activity was measured according to Bradley et al. [25]. Hepatic or colonic tissue was homogenized using assay buffer pH 6.0 (PBS, 50 mM, 0.5% hexadecyltrimethylammonium bromide and EDTA, 5 mM) and centrifuged at 1550 ×g/15 min (4°C). Supernatant was collected and centrifuged at 14.000 ×g/15 min (4°C). The sample was transferred (duplicate) to microplate and* ortho*-dianisidine solution (0.8 mg/mL) was added. After incubation (37°C/15 min), a solution of H_2_O_2_ (0.3%) was added. A new incubation was performed (37°C/10 min) and the reading was made at 460 nm. One unit of MPO was defined as the quantity that decomposes 1 *μ*mol of H_2_O_2_. The result was expressed as U·mg protein^−1^.

### 2.9. Measurement of Total GSH

GSH and GSSG were measured according to Tripple and Rogers [26], with slight modifications. First, total protein was precipitated, after centrifugation (190 ×g/10 min, at −20°C) with metaphosphoric acid (1 : 1). To obtain total GSH (tGSH), this supernatant was diluted in assay buffer (PBS 0.1 M, EDTA 5 mM, pH 7.4). Then, the homogenates were transferred to microplates, and the final volume (150 *μ*L) was completed with assay buffer. Afterwards, the reaction was started, upon addition of Reaction Mixture 1 (containing 5,5′-dithiobis-(2-nitrobenzoic acid), DTNB, 10 mM, and GR, 4.2 U/mL) and NADPH, 1% w/v.

To measure GSSG, the supernatant was diluted (1 : 50) in assay buffer, containing N-ethylmaleimide (NEM), and centrifuged (10,000 ×g/20 min at 4°C). This solution was incubated for 50 minutes to complete GSH complexation and removal. To exclude NEM, this supernatant with assay buffer were eluted through Sep-pak® Classic C18 cartridges. Afterwards, the eluent was transferred to microplate and Reaction Mixture 1 and NADPH,1% w/v, were added.

In both analyses, the absorbance was measured over 3 min at 412 nm with 30 s intervals. GSH was determined according to the following equation: GSH = tGSG − (GSSG/2). Results were expressed in nM·mg protein^−1^.

### 2.10. TNF-*α*, IL-10, and Interferon Gamma (INF*γ*) Levels

Cytokine production was quantified by Milliplex MAP Mouse Cytokine/Chemokine Panel (Merck Millipore), following the manufacturer's instruction. The results were expressed as pg·mg protein^−1^.

### 2.11. Statistical Analysis

Normality was assessed by the Kolmogorov-Smirnov test. Parametric variables were evaluated using the paired one-way analysis of variance (ANOVA), followed by Tukey's or Bonferroni's posttest. Student's* t*-test was performed just to confirm the disease in Mild colitis group. The Kruskal-Wallis test was used for assessing the nonparametric variables and corresponding* post hoc* analysis. Results were shown as mean ± standard error (SEM) for those with normal distribution and as median values and interquartile range for the nonparametric ones. Two-sided* p* value <0.05 was considered statistically significant. GraphPad® Prism version 5.0 for Windows software (San Diego, CA, USA) was used.

## 3. Results

For the sake of clarity, results are divided into three topics: general, colonic, and hepatic results.

### 3.1. General Results

#### 3.1.1. Mild Colitis and Supplementation Did Not Alter Body Weight, Food Intake, or Liver and Colon Weights

**Figure 1:**
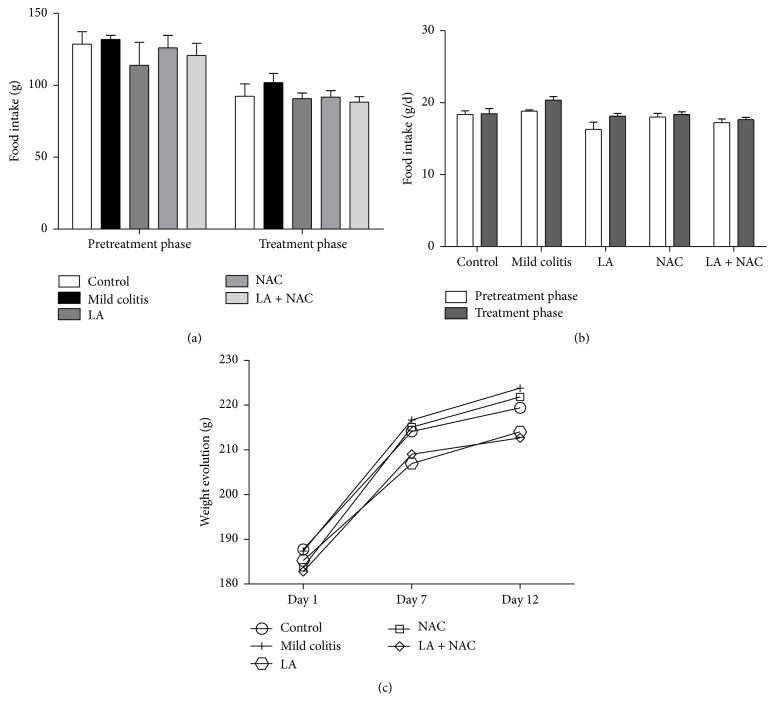
Food intake (g) (a); daily food intake (g/d) (b); weight evolution (g) (c) and daily weight gain (g/d) according to phase (pretreatment, PT; days 1 to 6, and treatment, T; days 7 to 12) and group (control; mild colitis; lipoic acid, LA;* N*-acetylcysteine, NAC; LA associated with NAC – LA + NAC).

In both phases (PT and T) of the study, DSS or supplementation by antioxidants did not induce effects on body weight or food intake patterns compared to the control group (Figures 1(a) and 1(b)). Similarly, body weight was unchanged over the evaluation period (Figure 1(c)). Absolute and relative liver and colon weights were unchanged (Table 1). Water ingestion modification was also not observed (data not shown).

#### 3.1.2. LA and NAC, but Not LA + NAC, Decreased Anemia and Leukocytosis Caused by Mild Colitis

**Figure 2:**
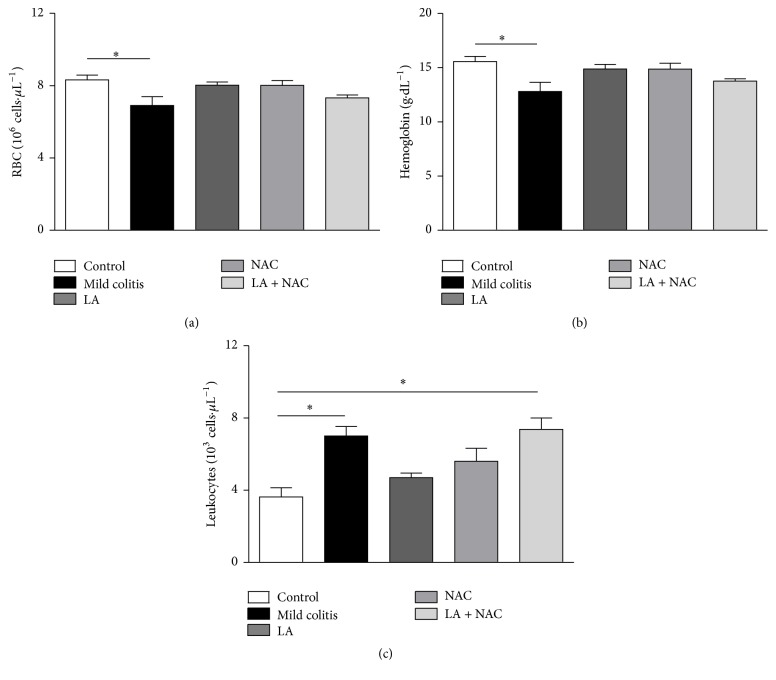
Red blood cells (RBC) (10^6^ cells·*μ*L^−1^) (a); hemoglobin (g·dL^−1^) (b); and leukocytes (10^3^ cells·*μ*L^−1^) (c), according to group (control; mild colitis; lipoic acid, LA;* N*-acetylcysteine, NAC; LA associated with NAC – LA + NAC). ^*∗*^ *p* < 0.05 (Tukey test).

Anemia in the mild colitis group was confirmed by a decrease in red blood cells (RBC) and hemoglobin (HB) (Figures 2(a) and 2(b)), since clinical changes such as rectal blood, diarrhea, and weight loss were not observed or did not show differences between the groups. No macroscopic change was observed after euthanasia (data not shown). All treatments increased the parameters (RBC, HB). However, LA and NAC alone ameliorated the typical UC inflammation, represented by a decrease in leukocytes, while the combination of LA + NAC did not show beneficial effects (Figure 2(c)).

#### 3.1.3. LA + NAC Increased Levels of Aminotransferases

The use of DSS and NAC did not cause a change in the available serological biomarkers, unlike LA supplementation, which promoted a decrease of globulin levels versus the mild colitis group (*p* < 0.01). However, this biochemical alteration did not exhibit physiological relevance, since albumin, the most important biomarker of hepatic function, remained statistically unaltered among the groups (Table 2). Moreover, it is important to observe that the combined action of LA and NAC on biomarkers of hepatic injury differed from the control (*p* < 0.05) and NAC (*p* < 0.05) groups. Compared to the NAC group, ALT and AST in the LA + NAC group were 2.2x and 2x higher, respectively. Despite the fact that these enzymes are not exclusive markers of liver damage, their increase in clinical situations such as heart disease and myopathies, when analyzed together with oxidative (Figure 7) and inflammatory (Figure 8) parameters, may be considered as a disruption of the liver metabolism balance. The other systemic biomarkers analyzed were not seen to be statistically significant.

### 3.2. Colonic Results

#### 3.2.1. LA or NAC Reduced Histological Damage on the Colon Induced by DSS

**Figure 3:**
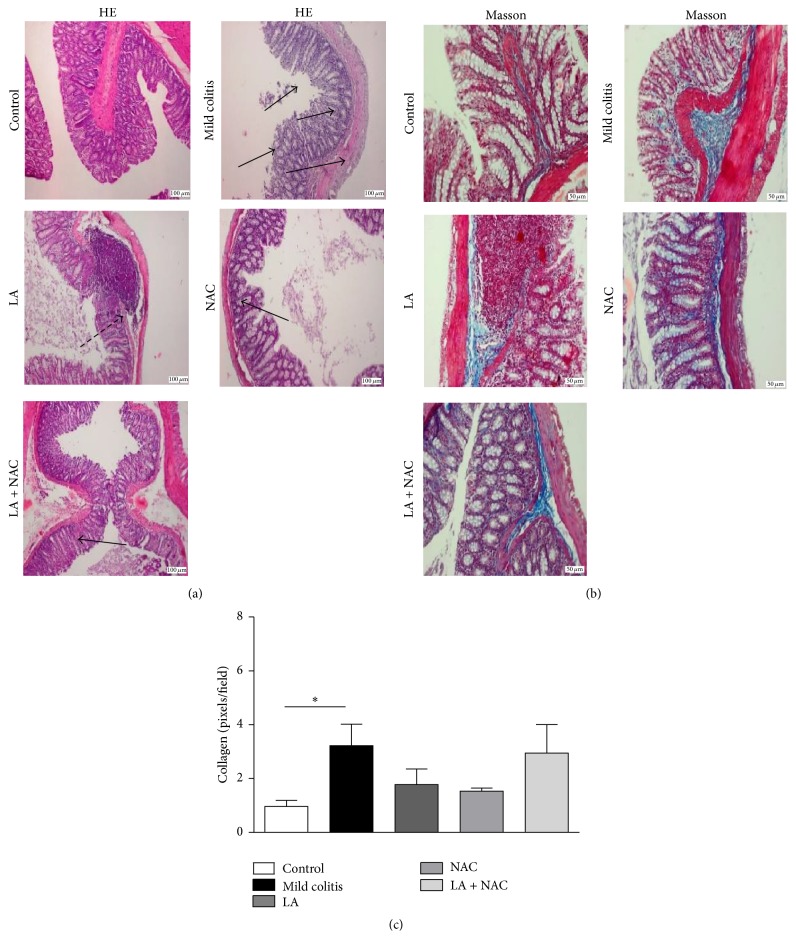
Hematoxylin and eosin staining (a) 50x magnification: arrows show the colonic lesions with neutrophil infiltration in mucosa and crypts' destruction; Masson trichrome staining (b) 100x magnification: the blue color shows areas with accumulation of mucous and collagen, especially on the lamina itself, submucosa, and between crypts. (c) Colonic collagen counts confirm fibrosis in the mild colitis group (control; mild colitis; Lipoic acid, LA;* N*-acetylcysteine, NAC; LA associated with NAC – LA + NAC). ^*∗*^ *p* < 0.05 (*Dunn's* test).

Samples from DSS-treated animals (mild colitis) showed higher histological damage (Figure 3(a)) than other groups. Although all the treated groups showed these changes, damage to the mucosal architecture was reduced when antioxidants were used, showing a protective effect of the antioxidants, when compared to the mild colitis group. Collagen deposition (Figure 3(b)), marked with a blue color, confirms the presence of fibrotic tissue in mild colitis (Figure 3(b)). LA and/or NAC treatments were able to decrease this deposition but were not sufficient to prevent mild UC lesions and their collagen counts were equal to the mild colitis and control groups (*p* > 0.05) (Figure 3(c)).

#### 3.2.2. Colonic Oxidative Damage Is the First Signal Observed in Mild Colitis

**Figure 4:**
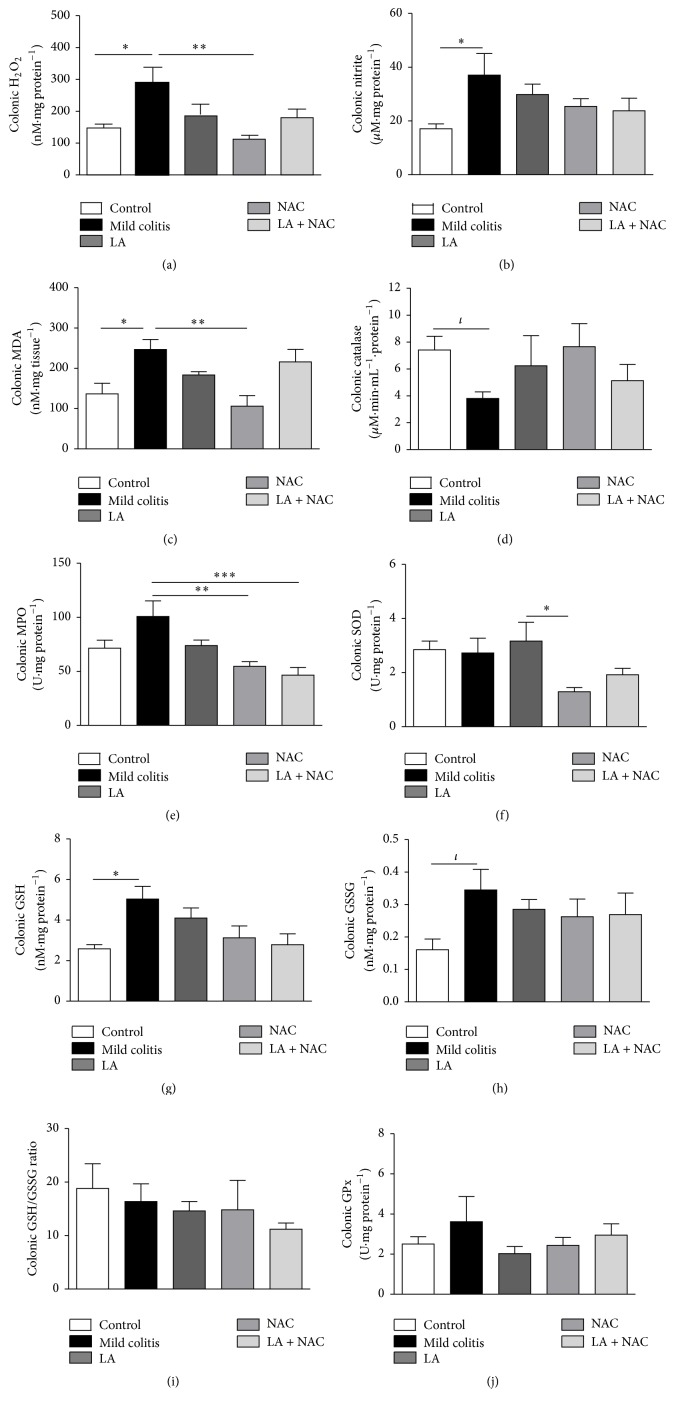
Colonic oxidative markers: hydrogen peroxide (H_2_O_2_) levels (a); nitrite levels (b); malondialdehyde (MDA) levels (c); catalase activity (d); myeloperoxidase (MPO) activity (e); superoxide dismutase (SOD) activity (f); reduced glutathione (GSH) levels (g); oxidized glutathione (GSSG) levels (h); GSH/GSSG ratio (i); and glutathione peroxidase (GPx) activity (j), according to group: control; mild colitis; lipoic acid, LA; N-acetylcysteine, NAC; LA associated with NAC − LA + NAC. ^*∗*^ *p* < 0.05 (Tukey test); ^*∗∗*^ *p* < 0.01 (Tukey test); ^*∗∗∗*^ *p* < 0.001 (Tukey test); ^*ι*^ *p* < 0.05 (*t*-test).

Oxidative damage represented by increased H_2_O_2_ (Figure 4(a)), nitrite (Figure 4(b)), and MDA (Figure 4(c)) levels, together with a decrease in CAT activity (Figure 4(d)), was already present in the mild colitis group and confirms the involvement of oxidative stress in the pathogenesis of UC. NAC was able to restore both H_2_O_2_ and MDA to levels equal to the control group and decreased MPO levels versus the mild Colitis group (*p* < 0.05), thereby confirming its higher antioxidant power relative to LA. Interestingly, NAC decreased SOD activity (Figure 4(e)) compared to the LA group. At the same time, it decreased colonic oxidative damage. In mild colitis, NAC activity may be maintained due to an increase in GSH (Figure 4(g)) and consequently GSSG (Figure 4(h)), as a response attempt of the body to oxidative damage. There were no alterations in the GSH/GSSG ratio (Figure 4(i)) and GPx levels for all groups (Figure 4(j)).

#### 3.2.3. Changes in Intestinal Cytokines Were Not Observed in Mild UC and LA + NAC Provoked Inflammation

**Figure 5:**
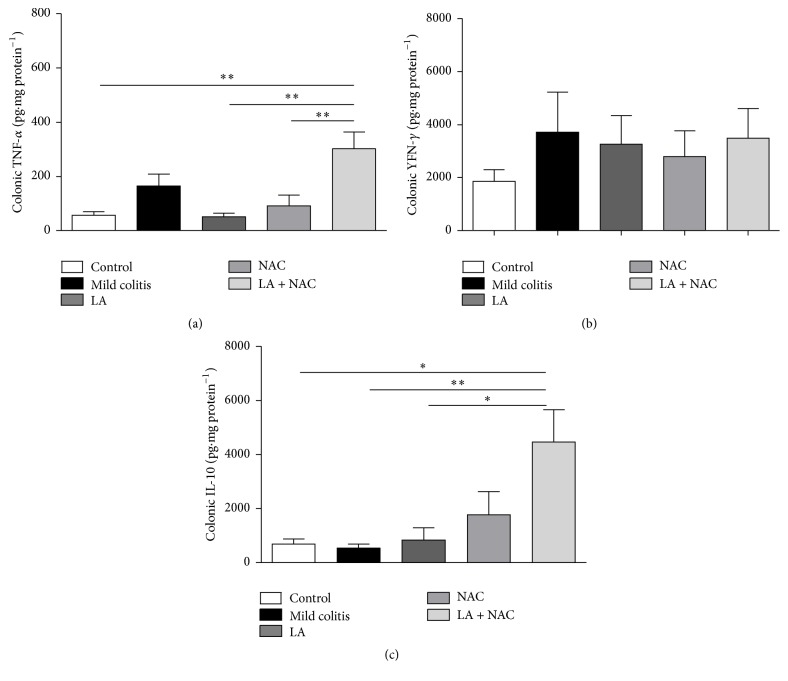
Colonic inflammatory markers: tumor necrosis factor alpha (TNF-*α*) (a); interferon gamma (INF-*γ*) (b); and interleukin 10 (IL-10) (c) levels according to group: control; mild colitis; lipoic acid, LA;* N*-acetylcysteine, NAC; LA associated with NAC – LA + NAC. ^*∗*^ *p* < 0.05, ^*∗∗*^ *p* < 0.01 (Tukey test).

Colonic inflammation represented by proinflammatory cytokines TNF-*α* and INF-*γ*, involved in innate immunity, and the anti-inflammatory cytokine IL-10, was not altered in the mild colitis group, compared to the control (Figures 5(a), 5(b), and 5(c)). However, LA + NAC promoted an increase in TNF-*α* (versus control, LA, and NAC groups) (Figure 5(a)) and IL-10 (versus all groups) (Figure 5(c)). Probably IL-10 increased to minimize the proinflammatory effects caused by TNF-*α*.

### 3.3. Hepatic Results

#### 3.3.1. Mild Colitis, LA, and/or NAC Did Not Cause Histological Alterations in the Liver

**Figure 6:**
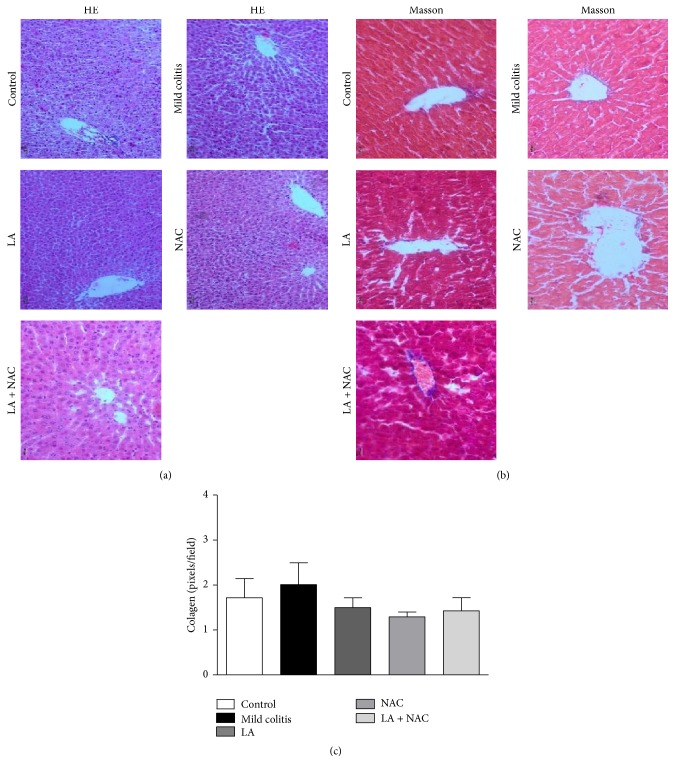
Hematoxylin and eosin staining (a) 50x magnification: Masson trichrome staining (b) 200x magnification. Hepatic collagen deposition (Figures 3(b) and 3(c)) confirms the absence of fibrotic tissue (control; mild colitis; lipoic acid, LA;* N*-acetylcysteine, NAC; LA associated with NAC – LA + NAC).

Biochemical and histological analyses (Table 2) suggest an absence of hepatic injury caused by DSS. However, despite the fact that the association between LA and NAC indicated concerning effects on this tissue, which can be observed by an increase in ALT and AST levels, only one morphological alteration could be identified: a periportal zone with disorganized hepatocyte cords (Figure 6(a)) without a necrosis area or collagen deposition (Figures 6(b) and 6(c)).

#### 3.3.2. LA and/or NAC Present Different Redox Modulations

**Figure 7:**
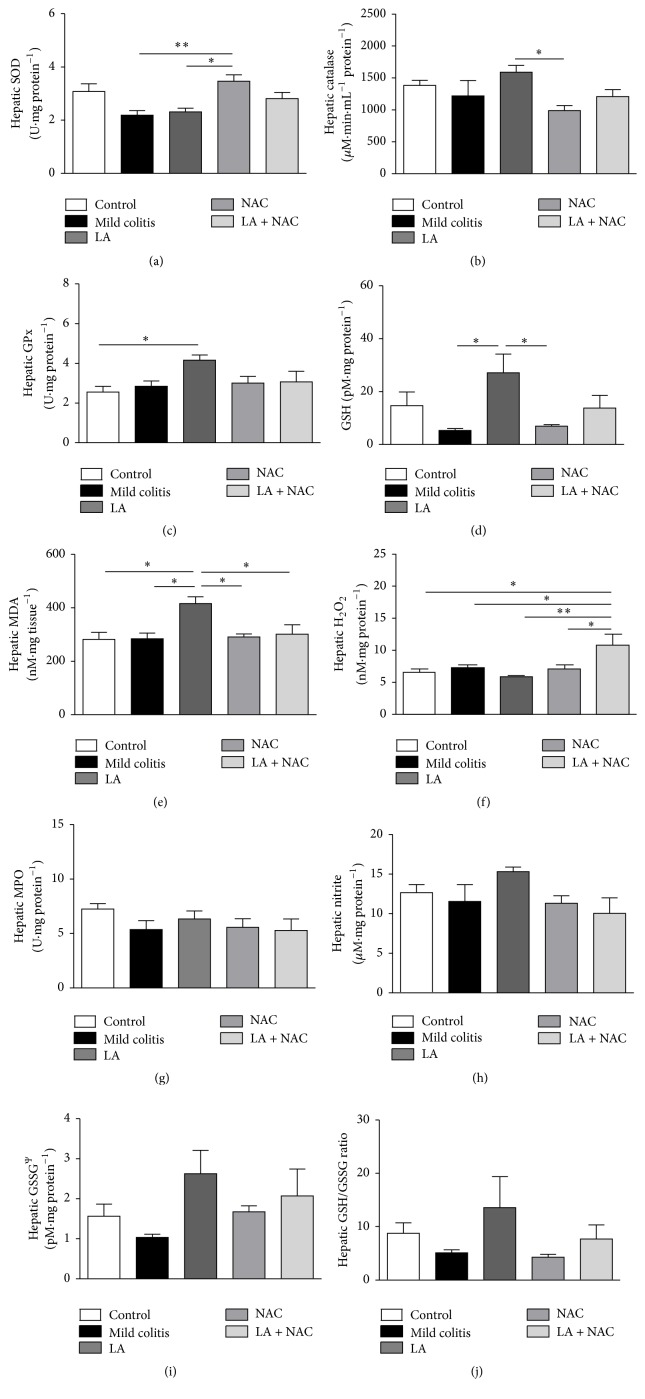
Hepatic redox markers: superoxide dismutase (SOD) activity (a); catalase activity (b); glutathione peroxidase (GPx) activity (c); reduced glutathione (GSH) levels (d); malondialdehyde (MDA) levels (e); hydrogen peroxide (H_2_O_2_) levels (f); myeloperoxidase (MPO) activity (g); nitrite levels (h); glutathione oxidized (GSSG) levels (i); and GSH/GSSG ratio (j), according to group: control; mild colitis; lipoic acid, LA; N-acetylcysteine, NAC; LA associated with NAC − LA + NAC. ^*∗*^ *p* < 0.05 (Tukey test). ^*∗∗*^ *p* < 0.01 (Tukey test). Ps.: GSSG (Kruskal-Wallis test).

DSS did not cause hepatic redox imbalance. NAC exhibited an improved SOD effect (increase) versus the LA group (Figure 7(a)), while LA increased CAT (versus NAC) (Figure 7(f)), GSH (versus mild colitis and NAC) (Figure 7(g)), and consequently GPx (versus control) (Figure 7(j)). These results confirm that different antioxidants act on redox imbalance* via* different pathways. However, as observed by biochemical analysis, LA + NAC acted as a prooxidant supplement, causing an increase in H_2_O_2_ (Figure 7(b)).

#### 3.3.3. LA + NAC Caused Inflammation in the Liver

**Figure 8:**
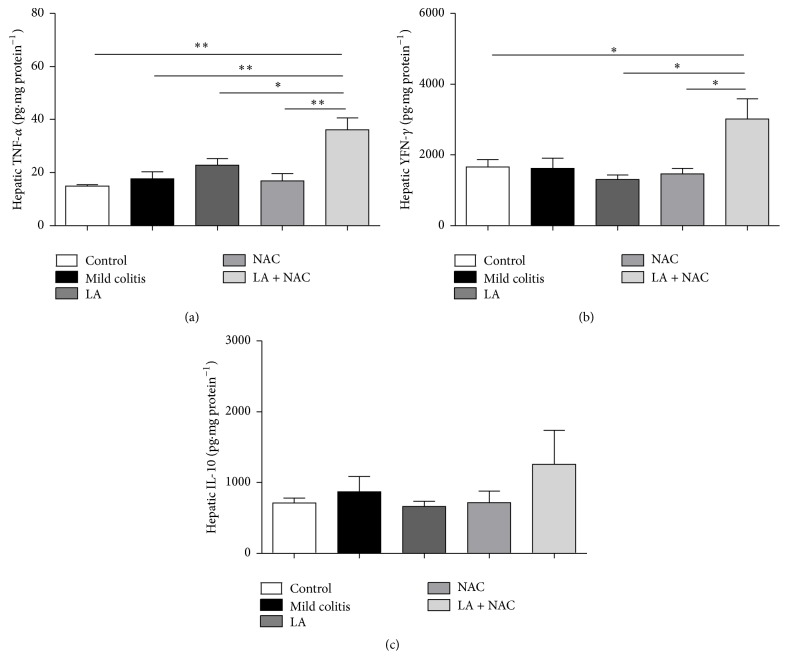
Hepatic inflammatory markers: tumor necrosis factor alpha (TNF-*α*) (a); interferon gamma (INF-*γ*) (b); and interleukin 10 (IL-10) (c) levels according to group: control; mild colitis; lipoic acid, LA;* N*-acetylcysteine, NAC; LA associated with NAC – LA + NAC. ^*∗*^ *p* < 0.05, ^*∗∗*^ *p* < 0.01 (Tukey test).

In the liver, the levels of cytokines were not modified in the mild colitis group compared to the control group (Figures 8(a), 8(b), and 8(c)). However, it is important to notice, in both tissues (colon and liver), the proinflammatory effects of LA associated with NAC (LA + NAC). In hepatic tissue, this combination provoked the increase of TNF-*α* and INF-*γ* levels, when compared to all groups (Figures 8(a) and 8(b)).

## 4. Discussion

Analyses of histology, oxidative stress, and inflammatory biomarkers were performed on colonic and liver tissues, in order to investigate the role of added antioxidants (NAC and/or LA) in controlling damage caused by DSS. In this context, we observed that redox imbalance was the first alteration in mild colitis; NAC was able to reduce oxidative stress and cell damage, DSS (2% w/w) did not cause hepatic modification, and LA + NAC increased inflammation in the colon and liver.

### 4.1. Colonic Injury and the Effects of Supplementation

Previous studies have provided compelling evidence for the association between DSS and different degrees of UC [27, 28], from mild colitis up to carcinogenesis, according to its continuous administration, at 2–5% w/v, for a short period of time (4–9 days). Moreover, histological, biochemical, and immunological alterations caused by DSS are similar to UC in humans [29]. The exact mechanism of colitis induction by DSS is not known, but it seems to involve dysfunctional macrophages, luminal bacterial alterations, and direct toxicity to epithelium [30].

The redox profile was the first biochemical parameter to be altered, before immunological changes, particularly in virtue of increased H_2_O_2_ and nitrite levels. Colonic injury typical of UC, represented by destruction of crypts and disorganization of intestinal structure, was observed in all groups that received DSS. Similar results were reported by other authors [31, 32]. However, LA and/or NAC supplementation could not prevent this damage, including collagen deposition (Figures 3(b) and 3(c)).

Even alone, LA has shown to exhibit negative effects in human studies. Wray et al. analyzing cardiovascular risk in elderly people observed that 600 mg/d of LA + Vit C (1000 mg) and Vit E (600 IU), 3x per week for 6 weeks, nullified positive effects on blood pressure, caused by exercise [33]. Additionally, McNeilly et al. [34], studying cardiovascular risk in obese patients with glucose intolerance, tested 1 g/d of LA for 12 weeks, with or without exercise, and detected no improvement on serum lipid profile and increased* oxLDL*. Showkat et al. [35] tested LA (600 mg), 30 minutes prior to iron administration in patients with chronic renal failure on hemodialysis, and observed an increase in F2 isoprostanes and lipid hydroperoxide, biomarkers of lipid peroxidation, thereby confirming cell disruption.

LA was able to scavenge H_2_O_2_ and decrease LP (MDA levels), but NAC was more effective, completely preventing the increase of these markers. NAC was also shown to be effective in chronic UC, induced by DSS (5% w/v for 5 days), as observed by Amrouche-Mekkioui and Djerdjouri [28]. According to these authors, NAC (150 mg·kg·d^−1^ for 45 days) decreased colitis symptoms, inflammation, cell apoptosis, and MPO and ^•^NO levels. Collectively, these results indicate the antioxidant and anti-inflammatory effects of NAC at different stages of UC.

^•^NO is a reactive molecule associated with UC progression, especially regarding toxic megacolon. In addition, ^•^NO reacts with O_2_^•−^, forming peroxynitrite, which causes LP and consequent ulcers in the colon mucosa. Both lesions are common in IBD [4]. ^•^NO production is catalyzed by the enzyme nitric oxide synthase (NOS). In inflamed tissue, such as the colon in UC, the inducible isoform of the enzyme (iNOS) is highly expressed in DSS-inflamed colons and the colon of UC patients [36]. This enzyme, present in its own colonocyte, may be responsible for the increase of nitrite levels in the mild colitis group.

F2-isoprostane is the best general indicator of nonenzymatic lipid peroxidation in complex biological systems [37]. However, in a recent systematic review on antioxidant therapy published by our group, we observed that the majority of the studies used MDA (identified directly by HPLC, or indirectly by thiobarbituric acid reactive substances, TBARS) to measure LP. In our study, a MDA assay was chosen.

In the present study, alterations in colonic SOD and GPx (Figures 4(f) and 4(j)) in the mild colitis group were not observed, which is similar to data reported by Akman et al. [38], who studied patients with active intestinal inflammation. On the other hand, the lower SOD activity observed in the NAC versus LA group, without evidence of increased H_2_O_2_ levels, confirms the major antioxidant power of NAC in our model.

Recently, antioxidant therapy for IBD has gained increased recognition [4]. However, SOD activity has been poorly investigated regarding LA and NAC administration, probably because SOD is an enzyme whose activity is modestly reduced, during tissue inflammation, unlike GPx2, a gastrointestinal-specific form of GPx [36], which is closely associated with H_2_O_2_ metabolism, and whose gene expression is downregulated in several experimental models of UC [39].

Our findings on elevated GSH and GSSG levels in the mild colitis group are different from the findings of Amrouche-Mekkioui and Djerdjouri [28]. This increase may be explained by the role played by GSH in inhibiting apoptosis signaling not only by scavenging intracellular ROS but also by inhibiting cytochrome c release from mitochondria and regulating the activity of redox-sensitive caspases [40]. In our results, the influence of oxidative stress on GSH cycling was confirmed by an increase of GSSG. However, the concomitant elevation of GSH levels did not cause a change in the GSH/GSSG ratio, the most important biomarker.

The GI tract is a major site for generation of prooxidants, whose production is primarily due to the presence of a plethora of microbes, food ingredients, and interactions between immune cells [15]. The enhanced production of reactive species is associated with chronic intestinal inflammation in the early stages of IBD. Their destructive effects on DNA, proteins, and lipids may contribute to initiation and progression of UC, causing several symptoms, such as loss of blood and anemia, carcinogenesis, hepatotoxicity, nephrotoxicity, and hypersensitivity [41]. Besides that, oxidative stress increases inflammation and stimulates activation of NF-kB, with consequent production of proinflammatory cytokines, chemokines, growth factors, and adhesion molecules, which cause inflammation and fibrosis, identified in our study by the increase of leukocyte levels and collagen deposition.

TNF-*α* has been shown to play a critical role in the pathogenesis of IBD and biological therapy with TNF-*α*-blockers has been used as a mainstream treatment for downregulating aberrant immune responses and inflammatory cascades [42]. Additionally, INF-*γ* is involved with overexpression of several chemokines, such as IFN-*γ*-inducible protein 10 and IFN-inducible T-cell *α* chemoattractant, in the intestinal mucosa for colitis induced in mice, and in UC patients [43]. On the other hand, IL-10, an anti-inflammatory cytokine, is required for regulating immune functions by promoting the widespread suppression of immune responses through its pleiotropic effects [44]. Imbalance in the production of these cytokines, such as TNF-*α*, plays a pivotal role in the signaling cascade of inflammatory pathways.

Guijarro et al. [45] in studying the effect of NAC plus mesalamine in UC patients also observed no changes in TNF-*α* plasma levels. It is possible that alterations were not identified because the model used in this study is for mild UC and upregulation of proinflammatory cytokines, such as IFN-*γ* and TNF-*α*, was observed more consistently in severe inflammation, such as colitis associated with carcinogenesis.

Unexpectedly, LA + NAC did not promote a beneficial action, even in increasing colonic IL-10 levels, which may be explained by the increase of leukocytes and dendritic cells, the latter responsible for its secretion and that are increased in colonic infiltrates [46]. IL-10 elevation has an anti-inflammatory response, especially in Th2 (T helper 2 lymphocytes) [47], related to autoimmune disorders such as UC [48]. In contrast to other studies [49, 50], a colonic increase of proinflammatory cytokines (Figures 5(a), 5(b), and 5(c)), despite the increase of leukocytes (Figure 2(c)), was not observed in the present study.

### 4.2. Hepatic Injury and the Effects of Supplementation

The extraintestinal manifestations of IBD are poorly explored by the scientific community. However, recent results have associated these manifestations to IBD activity [51] and the use of TNF-*α* inhibitors [52], exemplified by hepatobiliary manifestations, in terms of frequency and severity [53–55]. At the same time, these IBD complications remain underdiagnosed [56].

Despite the intimate connection between liver and colon, the oxidative and inflammatory alterations found in our model of mild colitis (2% of DSS, v/v, for 5 days) could have been insufficient to cause liver damage, unlike the results of Trivedi and Jena [57] and Farombi et al. [58]. However, when histological (Figure 6(a)), serological (Table 2), oxidative (Figure 7), and inflammatory (Figure 8) parameters were evaluated, hepatic damage in the LA + NAC group was identified.

Unlike hepatic ^•^NO (Figure 7(d)), H_2_O_2_ production was seen to be stimulated by the combination of LA + NAC (Figure 7(f)), while no modification in histology and collagen depositions was observed in this group.

Mitochondria and redox-active enzymes can generate O_2_^•−^ and H_2_O_2_ as byproducts in liver cells and these reactive molecules are increased under different conditions of chronic liver injury, caused by alcohol, xenobiotics, viral infections, nonalcoholic fatty liver disease, and others. Additionally, high concentrations of oxidative species, such as H_2_O_2_ and ^•^OH, induce hepatic stellate cell death and cause reduction of collagen deposition [59], which would explain the absence of changes in collagen deposition despite the higher levels of ALT and AST found in the serum of the LA + NAC group. Hepatic injury, whether acute or chronic, eventually results in an increase of serum concentrations of aminotransferases [60], suggesting stronger harmful action on the liver tissue, caused by the combination of these two antioxidants.

NAC supplementation led to an increase in hepatic SOD but without increasing H_2_O_2_ levels (versus mild colitis and LA), that is, improved redox status by activation of the antioxidant defense system. On the contrary, LA significantly increased CAT, GSH, and GPx, possibly due to increased oxidative stress observed in this group and confirmed by an elevation of MDA. Oxidative stress may be observed by GSSG levels, and although without statistical significance, these levels were approximately 61% higher than in the NAC group (Figure 7(i)).

Relative to lower CAT and GSH levels observed in the NAC group, these levels could be justified by the shorter period of supplementation. NAC protection against oxidative stress occurs by directing cysteine into the GSH synthesis pathway, with a consequent increase on the intracellular GSH content [16].

In a recent review, the authors showed that LA is generally administered associated with other substances and that this multiple therapy impairs the identification of the specific role of each component, raising difficulties in attributing beneficial, synergistic, or antagonistic effects [18]. In this context, El-Gowelli et al., also using an animal model, observed that LA plus cyclosporine, an immunosuppressant used routinely in UC treatment, aggravates colon damage. Pop-Busui et al. studying patients with type 1 diabetes observed similar noxious effects in diabetic patients, using LA plus allopurinol (xanthine oxidase inhibitor) and nicotinamide whose combination did not prevent the progression of cardiovascular autonomic neuropathy.

It is important to emphasize that the LA + NAC group received 200 mg·kg·d^−1^ (100 mg·kg·d^−1^ of each antioxidant), an amount much lower than the maximum limit established for safety in the oral delivery of LA (10x less) [61] and NAC (30x less) [62]. Taken together, these results cast doubt on the concept of the “universal antioxidant” given to LA.

## 5. Conclusions

**Figure 9:**
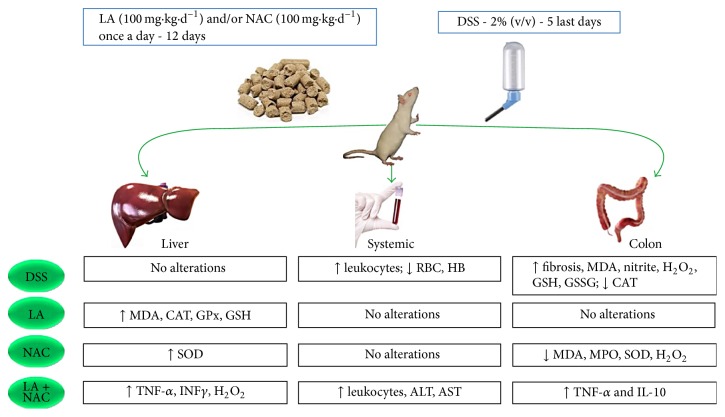
↑ = increased; ↓ = decreased; ALT = alanine aminotransferase; AST = aspartate aminotransferase; CAT = catalase; d = day; DSS = dextran sodium sulfate; GSH = glutathione reduced; GSSG = glutathione oxidized; H_2_O_2_ = hydrogen peroxide; HB = hemoglobin; IL = Interleukin; INF*γ* = interferon gamma; LA = lipoic acid; MDA = malondialdehyde; MPO = myeloperoxidase; NAC = N-acetylcysteine; RBC = red blood cells; SOD = superoxide dismutase; TNF-*α* = tumor necrosis factor alpha.

In our study, oxidative stress was the first biochemical manifestation of mild UC and happened before an increase in TNF-*α* and INF-*γ*. Additionally, NAC exhibits better antioxidant effects, especially regarding MDA and H_2_O_2_ levels. LA, administered daily, as a single dose increased hepatic MDA. LA + NAC increased oxidative and inflammatory profiles in the colon and liver (Figure 9).

In summary, our work provides evidence that the antioxidant and anti-inflammatory power of NAC involves not only the colon but also the liver. This fact confirms, for UC, the necessity to broaden the investigation to the liver, which is intimately connected to the colon.

The management of UC with alternative therapies is a large field of investigation and experimental colitis must mimic the human disease spectra. As presently shown, investigation on several tissues and organs is necessary, before a definite choice of a treatment can be made.

## Figures and Tables

**Table 1:** Absolute and relative weights of colon and liver for the different groups (mean ± SEM), according to group: control; mild colitis; lipoic acid, LA; N-acetylcysteine, NAC; LA associated with NAC – LA + NAC.

Tissue/Ratio	Control	Mild colitis	LA	NAC	LA + NAC
Colon (g)	2.54 ± 0.10	2.30 ± 0.14	2.21 ± 0.11	2.19 ± 0.15	2.02 ± 0.10
Colon/body weight ratios	0.01 ± 0.00	0.01 ± 0.00	0.01 ± 0.00	0.01 ± 0.00	0.01 ± 0.00
Liver (g)	8.74 ± 0.40	8.76 ± 0.28	8.57 ± 0.31	8.22 ± 0.39	8.40 ± 0.14
Liver/body weight ratios	0.04 ± 0.00	0.04 ± 0.00	0.04 ± 0.00	0.04 ± 0.00	0.04 ± 0.00

**Table 2:** Biochemical plasma analysis (mean ± SEM), according to group: control; mild colitis; lipoic acid, LA; *N*-acetylcysteine, NAC; LA associated with NAC – LA + NAC.

	Control	Mild colitis	LA	NAC	LA + NAC
Hb1Ac (%)^Ψ^	7.80 ± 0.15	7.97 ± 0.29	8.50 ± 0.40	8.13 ± 0.33	7.88 ± 0.28
Glucose (mg/dL)	189.00 ± 31.35	207.80 ± 38.77	196.00 ± 61.01	156.2 ± 23.66	176.40 ± 18.76
CRP (mg/dL)	2.22 ± 0.32	2.20 ± 0.16	1.90 ± 0.25	2.15 ± 0.30	2.18 ± 0.29
Calcium (mg/dL)	9.25 ± 0.31	8.90 ± 0.29	8.13 ± 0.90	8.68 ± 0.23	8.64 ± 0.41
TC (mg/dL)	76.00 ± 7.69	65.75 ± 7.20	70.50 ± 5.50	72.25 ± 5.45	64.33 ± 8.67
TG (mg/dL)	55.50 ± 14.56	63.25 ± 14.48	53.50 ± 0.71	54.25 ± 7.01	50.20 ± 19.83
LDL-C (mg/dL)	37.56 ± 14.37	30.32 ± 5.67	31.80 ± 3.60	35.90 ± 5.37	29.96 ± 5.70
HDL-C (mg/dL)	27.33 ± 2.26	24.50 ± 1.76	28.00 ± 2.00	25.50 ± 0.87	23.60 ± 3.17
AST (U/L)	167.50 ± 27.91	194.00 ± 20.72	297.75 ± 59.74	153.40 ± 20.34	359.75 ± 76.95^*αδ*^
ALT (U/L)	82.83 ± 5.83	104.25 ± 6.14	133.00 ± 24.78	85.25 ± 6.02	168.25 ± 38.60^*αδ*^
AP (U/L)	420.50 ± 33.84	345.00 ± 12.45	315.00 ± 66.24	302.40 ± 46.64	305.00 ± 53.97
TB (mg/dL)	0.12 ± 0.00	0.10 ± 0.02	0.10 ± 0.02	0.12 ± 0.01	0.10 ± 0.02
DB (mg/dL)^Ψ^	0.03 ± 0.00	0.02 ± 0.00	0.03 ± 0.00	0.02 ± 0.00	0.03 ± 0.00
IB (mg/dL)^Ψ^	0.10 ± 0.00	0.07 ± 0.02	0.08 ± 0.00	0.09 ± 0.01	0.08 ± 0.01
TP (g/dL)	5.24 ± 0.19	4.63 ± 0.48	4.20 ± 0.41	4.78 ± 0.17	4.66 ± 0.14
ALB (g/dL)	2.90 ± 0.27	2.28 ± 0.89	2.50 ± 0.46	2.74 ± 0.18	2.68 ± 0.15
GLOB (g/dL)	2.30 ± 0.09	1.95 ± 0.03	1.70 ± 0.15^*αα*^	2.04 ± 0.10	1.98 ± 0.10

Hb1Ac = glycosylated hemoglobin; CRP = C reactive protein; TC = total cholesterol; TG = triacylglycerol; LDL-C = low-density lipoprotein; HDL-C = high density lipoprotein; AST = aspartate aminotransferase; ALT = alanine aminotransferase; AP = alkaline phosphatase; TB = total bilirubin; DB = direct bilirubin; IB = indirect bilirubin; TP = total protein; ALB = albumin; GLOB = globulin; ^*α*^ *p* < 0.05 versus control; ^*αα*^ *p* < 0.01 versus control; ^*δ*^ *p* < 0.05 versus NAC (Tukey test). ^Ψ^Kruskal-Wallis and Dunn's test.

## Abbreviation List

ALB: Albumin
ALT: Alanine aminotransferase
ANOVA: One-way analysis of variance
AP: Alkaline phosphatase
AST: Aspartate aminotransferase
BHT: Butylated hydroxytoluene
CAT: Catalase
CD: Crohn's disease
CRP: C reactive protein
d: Day
DB: Direct bilirubin
DHLA: Dihydrolipoic acid
DNA: Deoxyribonucleic acid
DSS: Dextran sodium sulfate
DTNB: 5,5′-Dithiobis-2-nitrobenzoic acid
GI: Gastrointestinal
GLOB: Globulin
GPx: Glutathione peroxidase
GR: Glutathione reductase GR
GSH: Glutathione reduced
GSSG: Dlutathione oxidized
H_2_O_2_: Hydrogen peroxide
HB: Hemoglobin
Hb1Ac: Glycosylated hemoglobin
HDL-C: High-density lipoprotein
HE: Hematoxylin and eosin
HPLC: High performance liquid chromatography
IBD: Inflammatory bowel diseases
IB: Indirect bilirubin
IKK2: I*κ*B kinase-2
IL: Interleukin
INF*γ*: Interferon gamma
i.p.: Intraperitoneal
Keap 1: Kelch ECH associating protein 1
LA: Lipoic acid
LDL-C: Low-density lipoprotein
LP: Lipid peroxidation
MDA: Malondialdehyde
MPO: Myeloperoxidase
NAC: * N*-Acetylcysteine
NADP^+^: Nicotinamide adenine dinucleotide phosphate oxidized
NADPH: Nicotinamide adenine dinucleotide phosphate reduced
NEM: N-Ethylmaleimide
NF-kB: Nuclear factor kappa B
Nrf2: Nuclear factor (erythroid-derived 2)-like 2
PBS: Potassium phosphate buffer
PT: Pretreatment
RBC: Red blood cells
SEM: Mean ± standard error
SOD: Superoxide dismutase
T: Treatment
TB: Total bilirubin
TC: Total cholesterol
TG: Triacylglycerol
tGPx: Total GPx
Th2: T helper 2 lymphocytes
TNF-*α*: Tumor necrosis factor alpha
TP: Total protein
UC: Ulcerative colitis
Vit: Vitamin.

## Additional Points

*Highlights.* Oxidative stress occurs before inflammatory alterations in mild colitis.* N*-Acetylcysteine is a promising antioxidant toward alleviating ulcerative colitis. Lipoic acid and* N*-acetylcysteine improved oxidative biomarkers for colonic disease. The combination of lipoic acid and* N*-acetylcysteine causes important hepatic injury and colonic inflammation. Antagonist effects of antioxidants have been proven, when administered together in mild colitis.
